# Attenuation of p38α MAPK stress response signaling delays the *in vivo* aging of skeletal muscle myofibers and progenitor cells

**DOI:** 10.18632/aging.100802

**Published:** 2015-09-28

**Authors:** John Papaconstantinou, Chen Z. Wang, Min Zhang, San Yang, James Deford, Dmitry V. Bulavin, Naseem H. Ansari

**Affiliations:** ^1^ The Department of Biochemistry and Molecular Biology, The University of Texas Medical Branch, Galveston, TX 77551-06743, USA; ^2^ Institute for Research on Cancer and Ageing of Nice, INSERM, U1081-UMR CNRS 7284, University of Nice – Sophia Antipolis, Centre Antoine Lacassagne, Nice, France

**Keywords:** aging, gastrocnemius, progenitor cells, myofibers, p38α, juvenile protective factors

## Abstract

Functional competence and self-renewal of mammalian skeletal muscle myofibers and progenitor cells declines with age. Progression of the muscle aging phenotype involves the decline of juvenile protective factors *i.e.,* proteins whose beneficial functions translate directly to the quality of life, and self-renewal of progenitor cells. These characteristics occur simultaneously with the age-associated increase of p38α stress response signaling. This suggests that the maintenance of low levels of p38α activity of juvenile tissues may delay or attenuate aging. We used the dominant negative haploinsufficient p38α mouse (DN-p38α^AF/+^) to demonstrate that *in vivo* attenuation of p38α activity in the gastrocnemius of the aged mutant delays age-associated processes that include: a) the decline of the juvenile protective factors, BubR1, aldehyde dehydrogenase 1A (ALDH1A1), and aldehyde dehydrogenase 2 (ALDH2); b) attenuated expression of p16^Ink4a^ and p19^Arf^ tumor suppressor genes of the *Cdkn2a* locus; c) decreased levels of hydroxynonenal protein adducts, expression of COX2 and iNOS; d) decline of the senescent progenitor cell pool level and d) the loss of gastrocnemius muscle mass. We propose that elevated P-p38α activity promotes skeletal muscle aging and that the homeostasis of p38α impacts the maintenance of a beneficial healthspan.

## INTRODUCTION

Progressive age-associated loss of skeletal muscle mass and function are attributed to the decline of expression of such juvenile protective factors as BubR1, a mitotic checkpoint surveillance protein [1, 2], ALDH1A1 [3-6] and ALDH2, proteins that protect against oxidative stress [7]. These proteins maintain efficient and beneficial physiological tissue specific functions of both terminally differentiated tissues and their specific progenitor cell populations during early to mid-post-natal growth and development. At the same time, the appearance of p16Ink4a and p19Arf, proteins of the *Cdkn2a* tumor suppressor locus, corresponds with the decline of proliferative and regenerative capacity of adult progenitor cells, i.e. the irreversible growth arrest [8-13]. These age-associated changes occur simultaneously with the chronic up-regulation of p38MAPK (p38α) stress response signaling, thus suggesting that their state of chronic inflammation promotes the development of the aging phenotype (AP) [14-20].

Chronically elevated p38α activity is characteristic of a pro-inflammatory state that promotes expression of the physiologically complex AP. It is well established that p38α is the sole member of the p38MAPK family whose levels of activity play a key role in the promotion of senescence [14, 16, 18-22], as well as the regulation of myogenesis [17, 23]. This specificity is further indicated by the fact that myoblasts lacking p38α promote adult progenitor cell proliferation while restricting differentiation, thus resulting in an increased reservoir of progenitor cells [17].

The maintenance of low levels of p38α activity regulates the progression of the AP in multiple tissues, as indicated by the improved regenerative capacity of aged skeletal muscle progenitor cells treated with inhibitors of p38α/β activity [8, 9] and the prolonged capacity of aged pancreatic β-cells to proliferate in response to injury by streptozotocin [24]. In addition, attenuation of p38α activity has been shown to delay the expression of p6^Ink4a^, p19^Arf^, p15^Ink4b^, and p21^Waf^ tumor suppressor genes. The p38α-mediated regulation of myogenesis thus involves a balanced regulation of proliferation vs. terminal differentiation [25] and increased p38α activity promotes skeletal muscle aging. These studies support our hypothesis that attenuation, not ablation or overexpression of p38α [26-29], serves as a major mechanism that delays senescence and age-associated diseases [14].

In this study we propose to demonstrate the importance of the attenuation of p38α activity in delaying or attenuating the expression of proteins of the AP *in vivo*. To address this we used the dominant-negative p38α mouse (DN-p38α^AF/+^), a haplo-insufficient genetic model in which the specific attenuation of P-p38α delays the expression of aging characteristics [24]. We measured the alteration of expression levels of a group of *in vivo* aging markers that include juvenile protective factors whose functions decline and tumor suppressor genes whose functions increase with age, in order to demonstrate that their levels of expression are linked to the levels of p38α activity. Here, we focused on demonstrating that BubR1, ALDH1A1 and ALDH2 are beneficial juvenile protective factors whose activities decline during the transition to aging, and importantly, that this decline is attenuated in the physiological environment promoted by the decreased p38α activity of the DN-p38α^AF/+^ mutant. Secondly, we demonstrate that the attenuated expression of the AP markers, p16^Ink4a^ and p19^Arf^ in the aged DN-p38α^AF/+^ mouse suggest a delay in aging; thirdly that the age-associated loss of muscle mass and decline of progenitor cell population is delayed.

## RESULTS

The elevated levels of p38α activity (P-p38α), a characteristic of the chronic pro-inflammatory state of aging tissues, promotes the progression of the AP [14, 16, 22, 23]. Low levels of P-p38α activity of juvenile tissues are associated with a physiological environment that is beneficial for efficient tissue and progenitor cell functions whereas sustained, elevated P-p38α activities promote the expression of physiological markers of the AP [14, 31]. The levels of P-p38α thus serve to regulate the physiological characteristics of either juvenile or senescent tissues. To demonstrate the role of P-p38α activity in the progression of skeletal muscle progenitor cell and myofiber aging, we used the dominant negative p38α mouse (DN-p38α^AF/+^) in which the substitution of the Thr^180^ ➔ Ala and Tyr^182^ ➔ Phe attenuates p38α activity by eliminating the catalytic site phosphorylation and kinase activity of one allele [24].

### The level of P-p38α is attenuated in the gastrocnemius of aged DN-p38α^AF/+^ mice

The p38α^AF/+^ allele encodes a dominant negative p38α isoform such that the mutation of one allele is sufficient to specifically suppress p38α signaling *in vivo* [24]. Evidence of the physiological benefits of the attenuation of P-p38α activity is indicated by the demonstration that disruption of a single copy of the p38α gene is cardioprotective against ischemia-reperfusion [32] and that the p38α-mediated phosphorylation of HSP25 is significantly reduced in the aged (24mos) DN-p38α^AF/+^ mutant [24].

**Figure 1:**
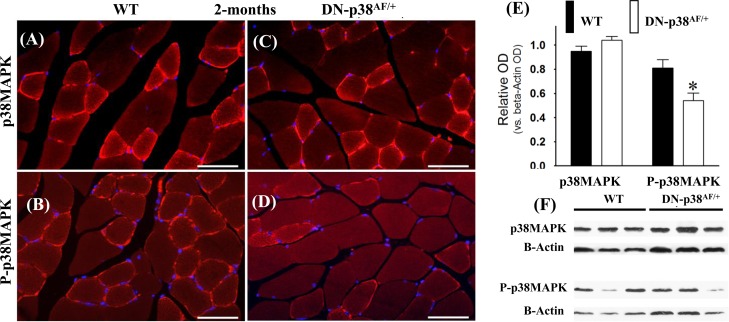
Expression of p38α and phospho-p38α in the gastrocnemius of young (2 mos old) wild type and DN-p38α^AF/+^ mice **(A-D)** Immunohistofluorescence analysis of the levels of p38α and P-p38α in cross sections of the gastrocnemius of young (2 mos) **(A, B)** WT and **(C, D)** DN-p38^AF/+^ mice. The red immunohistofluorescence depicts levels of p38α or P-p38α using antibodies specific for p38α and P-p38α; The blue immunofluorescence depicts DAPI stained nuclei; Scale bar = 50 μm. **(E)** A bar graph presentation of the western blot data in (**F**). The data in (**E**) are depicted as relative OD vs. β-Actin values of western blots. *p < 0.05 vs. corresponding WT. **(F)** Western blot (immunoblot) analysis of the levels of total p38α and total P-p38α in (**A**, **B**) young WT (2 mos) and (**C, D**) DN-p38α^AF/+^ mice.

**Figure 2:**
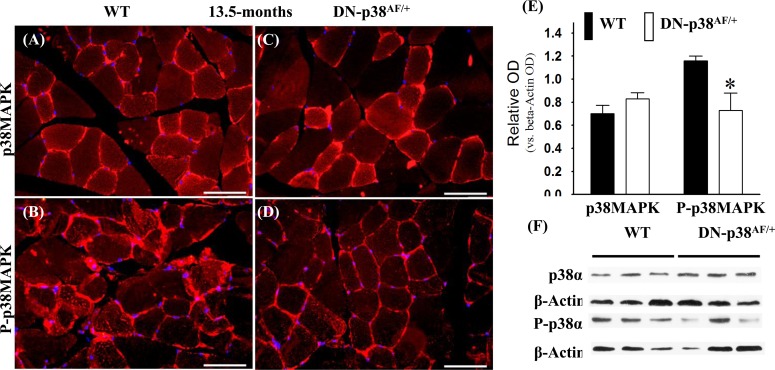
Expression of p38α and phospho-p38α in the gastrocnemius of middle-aged (13.5 mos old) wild type and DN-p38α^AF/+^ mice **(A-D)** Immunohistofluorescence analysis of the levels of p38α and P-p38α in cross sections of the gastrocnemius of middle aged (13.5 mos) **(A, B)** WT and **(C, D)** DN-p38α^AF/+^ mice. The red immunohistofluorescence depicts levels of p38α or P-p38α using antibodies specific to either p38α or P-p38α. The blue immunofluorescence depicts DAPI stained nuclei; Scale bar = 50 μm. **(E)** A bar graph presentation of the western blot data in (**F**). The data in (**E**) are depicted as relative OD vs. β-Actin values of Western blots. *p < 0.05, vs. corresponding WT. **(F)** Western blot (immunoblot) analysis of the levels of p38α pool and total P-p38α pool in (**A**, **B**) middle aged WT (13.5 mos) and (**C, D**) DN-p38^AF/+^ mice.

**Figure 3:**
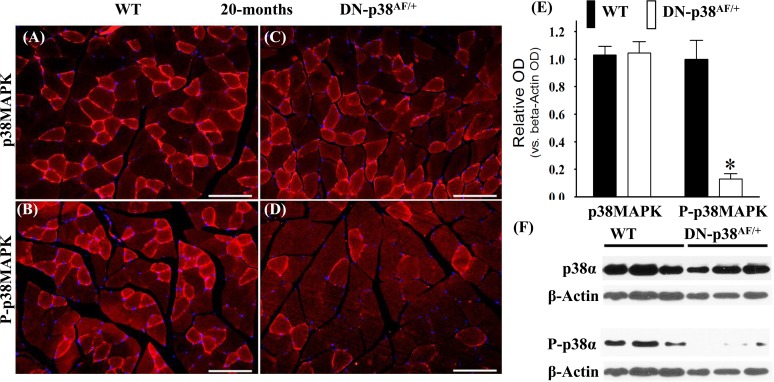
Expression of p38α and P-p38α in the gastrocnemius of aged (20 mos) wild type (WT) and DN-p38αAF/+ mice **(A-D)** Immunohistofluorescence analysis of the levels of p38α and P-p38α in cross sections of the gastrocnemius of aged (20 mos) **(A, B)** WT and **(C, D)** DN-p38α^AF/+^ mice; The red immunofluorescence depicts pool levels of total p38α or total P-p38α using antibodies specific to p38α and P-p38α. The blue immunohistofluorescence depicts DAPI stained nuclei; Scale bar = 50 μm. **(E)** A bar graph presentation of the western blot data in (**F**). The data in (**E**) are depicted as relative OD vs. β-Actin values of Western blots; *p < 0.05, vs. corresponding WT. **(F)** Western blot (immunoblot) analysis of the total p38α levels and total P-p38α pool in (A, B) aged WT (20 mos) and (**C, D**) DN-p38α^AF/+^ mice.

We conducted experiments to show that the overall p38MAPK pool levels are similar in the WT and DN-p38α^AF/+^ mice at all ages, whereas the levels of P-p38α are significantly lower in the mutant (Figures 1-3). The immunohistofluorescence of cross sections and immunoblots of the gastrocnemius extracts of WT and DN-p38α^AF/+^ mice show the protein pool levels of total p38α and P-p38α in young (Figure 1A-F), middle aged (Figure 2A-F), and aged (Figure 3A-F) mice. The data show that although the total pool levels of p38α are similar at all ages, the levels of P-p38α are significantly lower in the mutant than the corresponding age-matched controls.

### The attenuation of P-p38α activity delays the age-associated loss of gastrocnemius muscle mass

**Figure 4:**
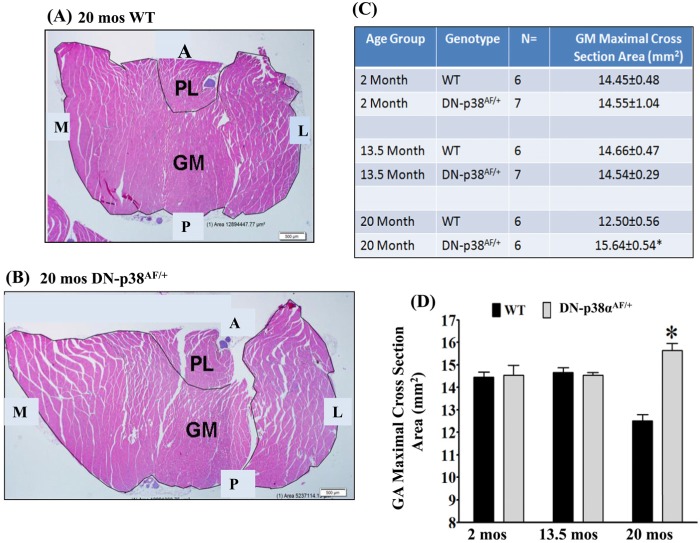
Cross sections of the gastrocnemius of 20-months old (A) WT and (B) DN-p38α^AF/+^ mice The cross sections suggest that the age-associated decrease of the diameter of the WT gastrocnemius is delayed in DN-p38α^AF/+^ mice. **(A)** shows a cross section of the gastrocnemius of aged WT (20 mos) mice; **(B)** shows a cross section of the gastrocnemius of aged DN-p38α^AF/+^ (20 mos) mice. (GM), gastrocnemius; (PL), plantaris. (**A**) anterior; (P) posterior; (M) medial; (L) lateral. **(C, D)** Maximal cross-sectional area of the gastrocnemius of young (2 mos), middle aged (13.5 mos) and aged (20 mos) mice. (**D**) A bar graph presentation of the data in (**B**).

Aged skeletal muscle undergoes sarcopenic atrophy and degeneration which is attributed to chronic inflammation [33-36]. The data in Figure 4 show that the loss of gastrocnemius muscle mass is delayed in the aged DN-p38α^AF/+^ mouse. Importantly the mean muscle diameters show a significant decrease in aged wild type (WT) mice, whereas in the corresponding age-matched N-p38α^AF/+^ mice the mean muscle diameters are significantly greater.

### The gastrocnemius progenitor cell population is not decreased in middle aged DN-p38α^AF/+^ mice

The age-associated decrease of the progenitor cell population is attributed to a reduced capacity of their self-renewal [8, 9, 17, 37, 38]. The data in Table 1 show that the number of progenitor cells associated with individual myofibers declines in the middle aged (13.5, mos old) WT gastrocnemius and that this decline is delayed in the corresponding age-matched DN-p38α^AF/+^ mutant. These results suggest that the decline of progenitor cell self-renewal capacity may have been delayed [24].

**Table 1:** A comparison of the gastrocnemius size, myofiber size, and satellite density of young vs. middle aged gastrocnemius in wild type vs. DN-p38α^AF/+^ hypomorphic C57B L/6 mice

Age (mos)	Genotype	N	Gastrocnemius Size (mm^2^)	Myofiber Size (μm^2^)	Satellite Density (nuclei/myofiber)
2	WT	3	3.90 ± 0.70	1600 ± 135	1.66 ± 0.22
2	ND-p38α ^AF/+^	5	3.62 ± 0.23	1379 ± 58*	1.67 ± 0.19
13.5	WT	3	3.86 ± 0.36	1572 ± 236	1.37 ± 0.13
13.5	ND-p38α ^AF/+^	5	3.37 ± 0.18*	1633 ± 194	1.93 ± 0.26**

Gastrocnemius size is expressed as mm^2^

Myofiber size is expressed as μm^2^ and

Satellite density is expressed as nuclei/myofiber

### BubR1 is a juvenile protective factor whose age-associated decline is delayed in the gastrocnemius of DN-p38α^AF/+^ mice

**Figure 5:**
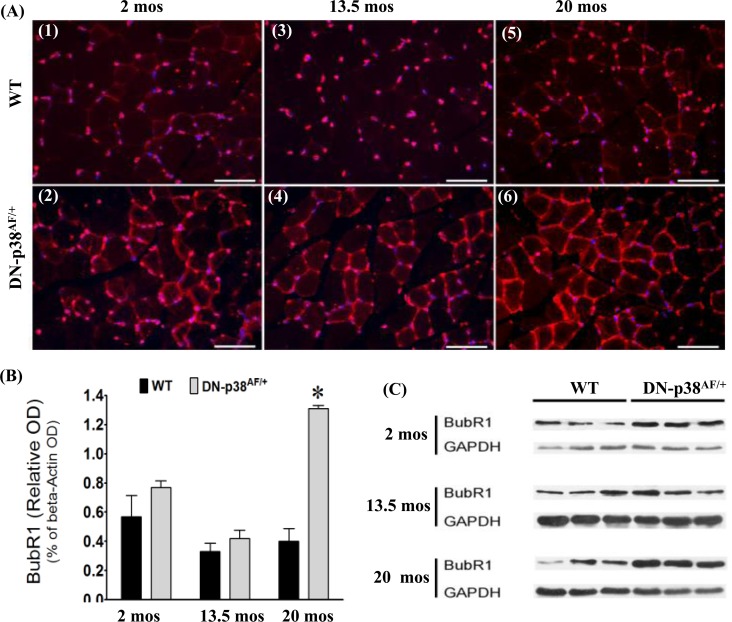
Expression of BubR1, a juvenile protective factor, in the gastrocnemius of young (2-3 mos), middle aged (13.5 mos) and aged (20 mos) WT and DN-p38^AF/+^ mice **(A: upper panel)** Levels of BubR1 expression in WT gastrocnemius decline with age. *(A1)* young (2 mos), *(A3)* middle aged (13.5 mos), and *(A5)* aged (20 mos); **(A: lower panel)** Levels of BubR1 expression in the gastrocnemius of DN-p38α^AF/+^ mice. *(A2)* young (2 mos); *(A4)* middle aged (13.5 mos); and aged *(A6)*. **(B)** A bar graph presentation of the western blot data in (**C**). Data are presented as relative OD vs. β-actin levels. *p < 0.05 vs. corresponding WT. **(C)** Western immunoblot analyses of the levels of BubR1 in young (2.0 mos), middle aged (13.5 mos) and aged (20 mos) WT and DN-p38α^AF/+^ mice. (GM, right leg). *p = 0.05 vs. WT of the same age.

The functions of the BubR1 juvenile protective factor involve mitotic checkpoint surveillance by regulating spindle assembly as well as ensuring accurate chromosomal segregation and the maintenance of genetic stability. Expression of the BubR1 gene declines in WT mice as they age [1, 2]. Furthermore, low levels of BubR1 in hypomorphic BubR1^H/H^ mice result in the development of multiple progeroid features, including short lifespan, sarcopenia, reduced stress tolerance, cachectic dwarfism, lordokyphosis, cataracts, loss of subcutaneous fat, and impaired wound healing [1, 2, 39-41]. Our studies indicate that BubR1 levels tend to be slightly higher in the gastrocnemius progenitor cells and myofibers of young (2-3 mos) and middle aged (13.5 mos) DN-p38α^AF/+^ mice whereas in aged (20 mos) mutant mice BubR1 levels are elevated ~3 fold compared to age-matched WT controls (Figure 5). To confirm that the decline of BubR1 protein levels diminish significantly in aged WT mice and that BubR1 expression is prolonged and elevated in the gastrocnemius of aged DN-p38α^AF/+^ mice (20 mos), we conducted Western blot analyses of gastrocnemius extracts. These results confirm that the expression of BubR1 declines in the middle aged and aged WT gastrocnemius whereas the level of expression is ~3 fold higher in the aged DN-p38α^AF/+^ muscle compared to the age-matched WT control (Figure 5C). We thus propose that the expression of BubR1 is elevated and prolonged in the gastrocnemius of these aged DN-p38α^AF/+^ mice. These results support our hypothesis that BubR1 is a juvenile protective factor whose functions are maintained in aged p38α^AF/+^ mice under conditions of attenuated P-p38α activity.

### Aldehyde dehydrogenase 1A1 (ALDH1A1) is a juvenile protective factor whose functions maintain cell integrity under conditions of oxidative stress

**Figure 6:**
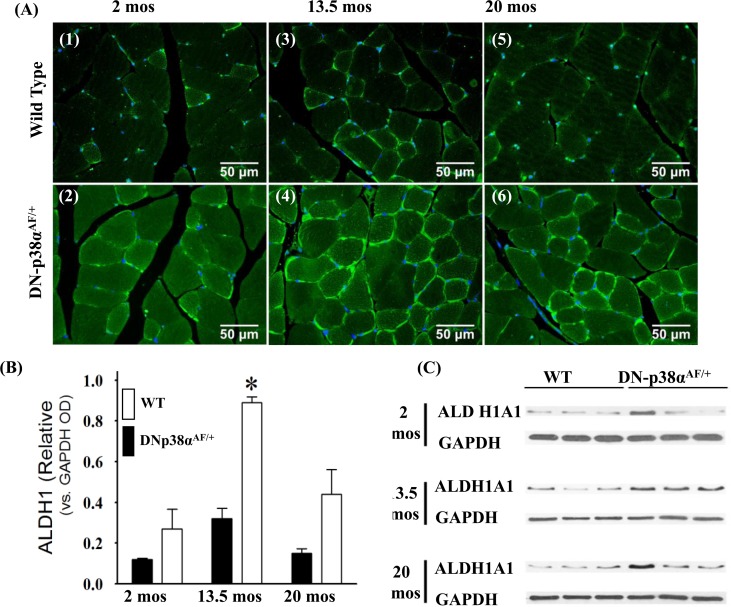
Expression of ALDH1A1, a juvenile protective factor, in the gastrocnemius of young (2 mos), middle aged (13.5 mos) and aged (20 mos) WT and DN-p38α^AF/+^ mice **(A: upper panel)** Levels of expression of ALDH1A1 in the gastrocnemius of WT mice. *(A1)* young (2.0 mos); *(A3)* middle aged (13.5 mos); *(A5)* aged (20 mos) mice. **(A: lower panel)** Levels of expression of ALDH1A1 in the gastrocnemius of DN-p38α^AF/+^ mice; *(A2)* young (2.0 mos); *(A4)* middle aged (13.5 mos), and *(A6)* aged (20 mos) mice. **(B)** A bar graph presentation of the western blot analyses of the data in (C). Data are presented as the relative OD vs. β-actin levels. **(C)** Western immunoblot analyses of the levels of ALDH1A1 in young (2.0 mos), middle aged (13.5 mos) and aged (20 mos) WT and DN-p38α^AF/+^ mice. (GM, right leg). *p = 0.05 vs. WT of the same age.

ALDH1A1 (cytoplasmic) and ALDH2 (mitochondrial) are ubiquitously distributed mammalian enzymes [42-46]. The functions of ALDH1A1 and ALDH2 include detoxification of the products of lipid peroxidation, such as 4-hydroxynonenal (HNE), acrolein and malondial-dehyde [47, 48], and the maintenance of cell integrity under conditions of oxidative stress (Figure 6). Of these, the cytotoxic lipid-derived aldehyde, HNE, propagates oxidative injury that promotes the AP. The data in Figure 6 show that the level of ALDH1A1 activity is significantly lower in the young, middle aged and aged WT gastrocnemius whereas its expression is elevated and prolonged throughout the life cycle of the DN-p38α^AF/+^ muscle. Our data show a significant elevated expression of ALDH1A1 in the middle aged mutant and although it decreases in the aged mutant, it remains ~2.5-fold higher than its age-matched control. The expression of ALDH1A1 in DN-p38α^AF/+^ mice is unique, however, in that it peaks at middle age and declines in the aged muscle. This pattern of expression also occurs in the WT muscle, but at a lower intensity.

**Figure 7:**
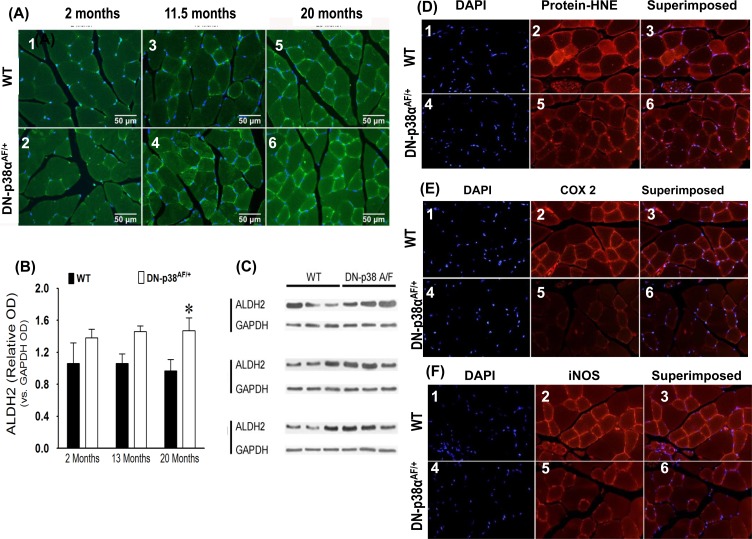
(A-C) Expression levels of ALDH2, a juvenile protective factor, by the gastrocnemius of young (2 mos), middle aged (13.5 mos) and aged (20 mos) WT and DN-p38α^AF/+^ mice **(A: upper panel)** The levels of expression of ALDH2 in the gastrocnemius of WT mice. *(A1)* young (2.0 mos); *(A2)* middle aged (13.5 mos); *(A3)* aged (20 mos) mice. **(A: lower panel)** The levels of expression of ALDH2 by the gastrocnemius of DN-p38α^AF/+^ mice; *(A4)* young (2.0 mos); *(A5)* middle aged (13.5 mos), and *(A6)* aged (20 mos) mice. **(B)** Bar graph presentation of the western blot analyses of the data in (C) presented as the relative OD vs. β-actin levels. *p < 0.05 vs corresponding WT. **(C)** Western immunoblot analyses of the levels of ALDH2 in young (2.0 mos), middle aged (13.5 mos) and aged (20 mos) WT and DN-p38α^AF/+^ mice. (GM, right leg). *p = 0.05 vs. WT of the same age. **(D-F)** Expression levels of protein-HNE complexes, COX2, and iNOS by young (3 mos) WT and DN-p38α^AF/+^ mice. **(D)** The level of expression of protein-HNE in the gastrocnemius of young (3mos) WT mice DN-p38α^AF/+^ mice *(D4-6)*. **(E)** The level of expression of COX2 in the gastrocnemius of young (3mos) WT mice *(E1-3)* and DN-p38α^AF/+^ mice *(E4-6)*. **(F)** The level of expression of iNOS in the gastrocnemius of young (3mos) WT *(F1-3)* and DN-p38α^AF/+^ *(F4-6)* mice.

We propose that the prolonged overexpression of ALDH1A1: a) ameliorates oxidation-induced toxicity of HNE modification [6, 48]; b) is associated with progenitor cell longevity and high myogenic capacities of human skeletal muscle cells [5] and c) is a beneficial juvenile protective factor whose functions diminish with age and whose elevated expression throughout the life cycle of the DN-p38^AF/+^ mouse may impart protection against oxidative stress as indicated by the significantly lower level of protein-HNE adducts (Figure 7D).

### Aldehyde dehydrogenase 2 is a mitochondrial specific juvenile protective factor whose expression is elevated throughout the life cycle of the DN-p38α^AF/+^ mouse

ALDH2 is located in the mitochondrial matrix and provides critical shielding from endogenous and exogenous damaging agents [7]. It and ALDH1A1 are responsible for the detoxification of biogenic and xenogenic aldehydes [7]. The functions of ALDH2 include detoxification of acetaldehyde, short chain aliphatic aldehydes, and some aromatic and polycyclic aldehydes [44]. Overexpressing or enhancing its activity ameliorates many of the deleterious effects of aldehydes and provide better protection against acute and chronic injuries induced by alcohol toxicity or oxidative stress. The levels of ALDH2 and ALDH1A1 are higher through-out the life-cycle of the DN-p38α^AF/+^mouse (Figures 6 and 7A-C) suggesting that the mutant is resistant to oxidative stress.

### The levels of protein-HNE adducts, COX2 and iNOS are attenuated in DN-p38α^AF/+^ mice

Our data show that the levels of protein-HNE adducts, COX2 and iNOS are lower in the young (3 mos) mutant (Figure 7). These results suggest that the beneficial juvenile protective factors work against oxidative stress and are established in young DN-p38α^AF/+^ mice as indicated by lower levels of protein-HNE complexes (Figure 7D). The resistance of DN-p38α^AF/+^ mice to oxidative stress is emphasized by the observation that the activities of iNOS and COX2 as well as the levels of protein-HNE adducts are all reduced (Figure 7E, F).

Interestingly, this occurs in young (3 mos) mice suggesting that this physiological characteristic appears in early juvenile tissues and may extend throughout the life-cycle of these mice.

### Expression of the p16^Ink4a^ cell cycle inhibitor is attenuated in the gastrocnemius of DN-p38α^AF/+^ mice

**Figure 8:**
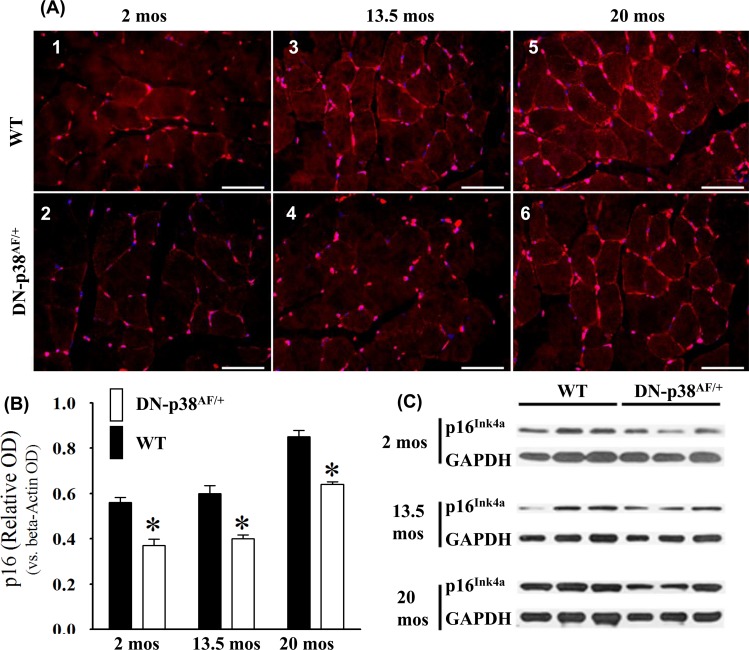
Expression of the p16^Ink4a^ tumor suppressor gene by the gastrocnemius of young (2 mos), middle aged (13.5 mos) and aged (20 mos) WT and DN-p38α^AF/+^ mice **(A: upper panel)** The levels of expression of the p16^Ink4a^ gene are significantly higher throughout the lifespan of WT mice compared to corresponding age matched DN-p38α^AF/+^ mice. Data for WT mice are depicted in *(A1)* young (2.0 mos); *(A3)* middle aged (13.5 mos); *(A5)* aged (20 mos); **(A: lower panel)** Data for DN-p38α^AF/+^ mice are depicted in *(A2)* young (2 mos); *(A4)* middle aged (13.5 mos) and aged *(A6)* aged (20 mos). **(B)** Bar graph presentation of the Western blot analyses of (C). *p = 0.05 vs. WT of the same age. **(C)** Western blot analyses of the level of expression of p16^Ink4a^ in gastrocnemius of young (2 mos); middle aged (13.5 mos) and aged (20 mos) WT and DN-p38α^AF/+^ mice.

The age-associated loss of skeletal muscle progenitor cell replicative capacity is a major problem in animal models and humans. The increased expression of p16^Ink4a^ is a well-established senescence-promoting signaling protein [49]. Expression of p16^Ink4a^ is higher in young WT gastrocnemius and increases significantly in aged mice. On the other hand, the p16^Ink4a^ expression by the DN-p38α^AF/+^ mice is lower than that of WT mice throughout their life cycle (Figure 8). This is consistent with the observation that the BubR1^H/H^ insufficient mice exhibit high levels of p16^Ink4a^ and that upon clearance of p16^Ink4a^ positive cells, characteristic pathologies of premature aging are attenuated [3]. Our results suggest that the attenuation of p16^Ink4a^ expression is an indication of delayed aging in the progenitor cells and myofibers of the aged DN-p38α^AF/+^ gastrocnemius.

### Expression of the p19^Arf^ cell cycle inhibitor is attenuated in gastrocnemius of aged DN-p38α^AF/+^ mice

**Figure 9:**
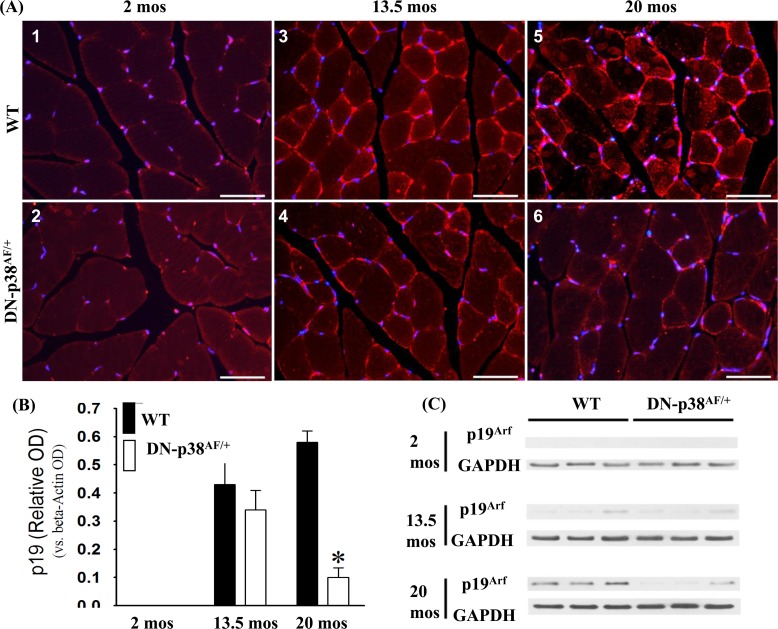
Expression of the p19^Arf^ tumor suppressor gene in the gastrocnemius of young (2 mos), middle aged (13.5 mos) and aged (20 mos) WT and DN-p38α^AF/+^ mice **(A: upper panel)** The levels of expression of the p19^Arf^ tumor suppressor gene are significantly higher throughout the life span of WT mice compared to corresponding age-matched DN-p38α^AF/+^ mice. Data for WT mice are depicted in *(A1)* young (2 mos): *(A3)* middle aged (13.5 mos); *(A5)* aged (20 mos) WT mice; **(A: lower panel)** Data for DN-p38α^AF/+^ mice are depicted in *(A2)* young (2 mos); *(A4)* middle aged (13.5 mos) and aged *(A6)* aged (20 mos) mice. **(B)** Bar graphs presentation of the Western blot analyses in **(C)**. Data are presented as relative OD vs. β-actin levels. *p < 0.05 vs WT of the same age. *p = 0.05 vs. WT of the same age. **(C)** Western blot analyses of the level of expression of p19^Arf^ in gastrocnemius of young (2 mos); middle aged (13.5 mos) and aged (20 mos) WT and DN-p38α^AF/+^ mice.

Expression of the p19^Arf^ gene is associated with age-related irreversible growth arrest, and its expression is elevated in aging cells *in vivo* as well as in culture [39, 50, 51]. Our data show that its expression is barely detectable in young gastrocnemius and increases significantly in middle aged and aged WT C57BL/6 mice (Figure 9). Our observations that p19^Arf^ expression is attenuated in the gastrocnemius of the young, middle-aged and aged DN-p38α^AF/+^ mice are consistent with observations of others who have shown that its expression is elevated in senescent cells in culture [31, 51-54]. The demonstration that the attenuation of p38α affects p19^Arf^ expression in our model provides important information regarding its role in age-related muscle atrophy [37. 53, 54].

## DISCUSSION

Our studies show that attenuation of the age-associated elevated level of P-p38α is linked to the delay of progression of the AP; that the p38α pathway serves as a major center for the distribution of signals that regulate expression of markers of aging.

The chronic elevated level of p38α signaling suggests that aging tissues are in a state of chronic inflammation [14-16, 18, 19, 22, 23] and establishes a physiological environment that supports the decline of juvenile protective factors and their beneficial functions. Our proposal that aging of the gastrocnemius progenitor cells and myofibers is delayed by reducing the levels of P-p38α activity in the DN-p38α^AF/+^ mouse is supported by the demonstration that its attenuation reduces the level of activation of cell cycle inhibitors of the *Cdkn2a* and *Cdkn2b* tumor suppressor loci in multiple organs (kidney, spleen, lung, liver) [24]. Furthermore, our proposal is consistent with the demonstration that attenuation of P-p38α delays the loss of proliferative and regenerative capacity of the pancreatic β-cells of aged DN-p38α^AF/+^ mice [24] and confers cardio-protection against ischemia-reperfusion [32].

**Figure 10:**
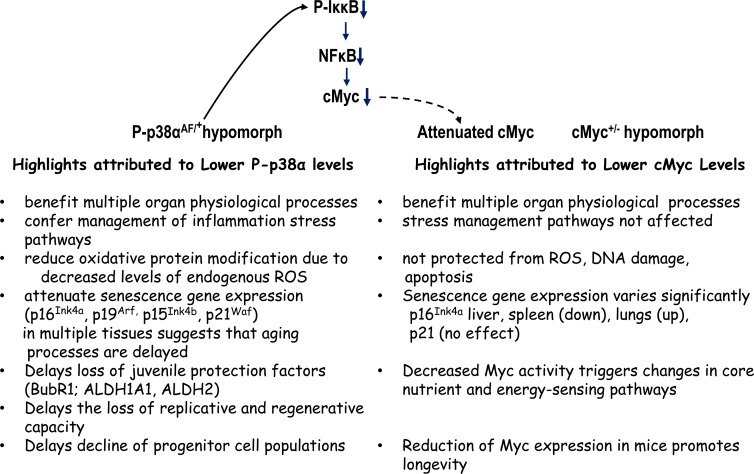
A summary and comparison of the physiological characteristics (phenotypes) of the DN-p38α^AF/+^ and cMyc^+/−^ hypomorphs The physiological consequences of attenuation of p38α or cMyc signaling activities suggest that the levels of their signaling activities play a key role in the delay of progression of the aging phenotype. The model above suggests that the DN-p38^AF/+^ mice, through their attenuation of phosphorylation of IκκB may decrease the NFκB activation of cMYC thereby contributing to the delay of the aging phenotype attributed to the cMYC^+/−^hypomorph. The studies concerning these hypomorphs emphasize the attenuation of two critical physiological centers of distribution of signals that promote the progression of the aging phenotype, i.e., oxidative stress, inflammation, various juvenile protective factors and tumor suppressors. Both studies stress the importance of attenuation, not ablation, of the activities in the regulation (homeostasis) of these signaling distribution centers.

The reduced expression of cMYC in a haploinsufficient mouse (cMYC^+/−^) results in an increased lifespan and has multiple beneficial effects on healthspan [30]. The results of our studies are consistent with these observations in that the overall progression of aging is delayed in the haploinsufficient p38α^AF/+^ model (Figure 10). Both of these studies emphasize the importance of homeostasis of two major distribution centers of stress response pathways and that both haploinsufficient p38α^AF/+^ and cMyc^+/−^ models exhibit multiple characteristics of beneficial healthspan. The pathway in Figure 10 suggests that the p38α^AF/+^ model may attenuate cMYC and thus exhibit some of the beneficial characteristics of the cMyc^+/−^ model.

The decline of juvenile protective factors in aging tissues compromises the maintenance of a healthy physiological environment [1, 2, 40, 41]. We propose that the chronic pro-inflammatory state and sustained elevated activity of the P-p38α pathway plays a major role in promoting the AP. Our studies suggest that BubR1 is a beneficial juvenile protective factor whose age-associated decline is delayed by the attenuation of P-p38α activities. Although these changes occur simultaneously with the chronically elevated P-p38α activity, interactions between BubR1 and P-p38α activity are not known.

Our proposal that BuBR1 is a juvenile protective factor is based on its role in “faithful segregation of replicated chromosomes which is essential for genetic stability [2]” and on the fact that its deficiency causes early onset of aging-associated phenotypes and infertility in mice. These results strongly support our hypothesis that BubR1 is an important juvenile protective factor whose prolonged expression in DN-p38α^AF/+^ mice delays their aging.

The mechanism of the age-associated decline of BubR1 is linked to the attenuation of SIRT2, a member of the NAD^+^-dependent deacetylases and chronic, elevated p38α activity [55]. SIRT2, for example, a) regulates BubR1 stability through deacetylation of its lysine 668 (K668) thereby inhibiting its ubiquitination and subsequent degradation; b) overexpression of SIRT2 extends both the mean and maximum lifespan of BubR1 ^(H/H)^ mice; c) reversal of the age-associated decline of NAD^+^ restores BubR1 protein levels. These studies have provided a major breakthrough in understanding the mechanism of its decline in aging tissues [55]. While the involvement of a SIRT2-p38α interaction is not known, our observation that BubR1 levels are higher in the aged (20 mos) DN-p38^AF/+^ mice does suggest such an interaction.

ALDH1A1 and ALDH2 serve to shield against damage due to oxidative stress. Their beneficial juvenile protective factor functions are suggested by reports that their overexpression ameliorates oxidation-induced toxicity of HNE-modified proteins [3, 4] and is associated with skeletal muscle progenitor cell longevity and high myogenic capacities [5]. However, chronic oxidative stress down regulates ALDH1A1 and ALDH2 with a concomitant increase in HNE-induced toxicity and promotion of the senescence phenotype. Thus, the decreased capacity to remove 4-HNE by both ALDH1A1 and ALDH2 is a proposed mechanism of aging [7, 56]. We thus propose that ALDH expression patterns in gastrocnemius progenitor cells of DN-p38α^AF/+^ mice, imparts myogenic potential and possibly resistance to oxidative stress.

The overall elevated levels of ALDH1A1 and ALDH2 and lower level of HNE-modified proteins of the gastrocnemius of DN-p38α^AF/+^ mice suggests that a physiological environment, protective against oxidative stress, is established throughout the life cycle of these animals and may thus slow down their aging processes. Our results suggest that limiting p38α signaling maintains the expression of ALDH1A1 and ALDH2 thereby attenuating free radical induced aging. Increasing their catalytic activity provides a novel effective means to reduce oxidative stress-induced cell and organ dysfunction and therefore supports health-span.

### The aging markers of the Cdkn2a tumor suppressor locus

Our studies suggest that the decreased level of P-p38α activity in the p38αAF/+ gastrocnemius attenuates the age-associated induction of multiple cell cycle inhibitors of the Cdkn2a and Cdkn2b loci [24]. The delay in their expression occurs in multiple tissues of the DN-p38αAF/+ mouse suggesting a simultaneous delay of the irreversible growth arrest and the senescence phenotype [24]. Our observation of the delayed aging of the gastrocnemius is consistent with a potentially global beneficial effect of this mutation on the maintenance and renewal of multiple tissues.

p38α has been identified as the specific member of the p38MAPK family (p38α, β, γ, δ) that regulates progenitor cell self-renewal. The elevated level of progenitor cells in the gastrocnemius of the middle aged DN-p38α^AF/+^ mutant suggests that self-renewal is retained by the mutant and is thus consistent with the observation that self-renewal is a function of the level of P-p38α activity, further emphasizing the importance of regulation of p38α homeostasis.

Pharmacological inhibition of the p38α pathway has been shown to reverse the defective self-renewal of the aged progenitor cells [8, 9], thereby mimicking the consequences of genetic inhibition of p38α (our DN-p38α^AF/+^). In one study it was demonstrated that inhibition of p38α/β in cultured aged skeletal muscle progenitor cells rejuvenates their potential for self-renewal, serial transplantation and the repair of damaged muscles of aged mice [8]. Bernet *et al* further showed that the basic cell autonomous loss of progenitor cell self-renewal is associated with the FGFR-1 mediated regulation of p38α/β signaling [9]. Other reports indicate that p38α inhibitors alleviate the symptoms of various p38-associated diseases such as cardiac remodeling of post myocardial infarction [32, 58]; neurodegenerative diseases such as Alzheimer's Disease [59], Amyotrophic lateral sclerosis [60], and inflammatory bowel disease [61]. Consistent with these findings, our studies suggest that this loss of function may be overcome *in vivo* by the attenuation of P-p38α levels, thereby decreasing the levels of expression of its downstream targets. We further contend that the *in vivo* attenuation and control of P-p38α activity will prolong self-renewal of the gastrocnemius progenitor cells and their proliferation and wound-healing capacity may be maintained in middle-aged (10-12 mos) and aged (24-26 mos) DN-p38α^AF/+^ mice.

## Conclusion

Our studies suggest that failure to regulate p38α homeostasis is an important factor in promoting aging and age-associated diseases. This is emphasized by the fact that ablation of p38α is embryonic lethal and that the overexpression of p38α activity accelerates aging [28, 62, 63]. We propose that the attenuation (but not ablation or overexpression) of p38α delays the progression of the aging phenotype and development of the irreversible growth arrest by prolonging expression of juvenile protective factors in aged tissues [64].

Musculoskeletal syndromes are major health problems that afflict the elderly of all societies. A certain percentage of our elderly will experience such age-associated diseases as cancer, cardiovascular disease, arthritis, etc., but practically all elderly worldwide will develop musculoskeletal syndromes such as sarcopenia and muscle atrophy. Our observations provide evidence that the attenuation of p38α activity may alleviate chronic inflammation and attenuate or delay the aging of skeletal muscle, *in vivo*.

## METHODS

### The DN-p38^AF/+^ mouse colony

Breeding pairs consisting of DN-p38α^AF/+^ males and females were provided by Dimitri V. Bulavin. The construction of these mutants has been described [24]. Our DN-p38α^AF/+^ colony was initiated by crossing DN-p38α^AF/+^ males and females and was maintained at the University of Texas Medical Branch in accordance with UTMB-IACUC protocols. This breeding protocol results in heterozygous progeny which were maintained for experimental use and for continuation of the colony. The wild type progeny were maintained for controls while the homozygous progeny are embryonic lethal. Genotyping of the mice was determined as described [24]. The University of Texas Medical Branch is accredited by the American Association for the Accreditation of Laboratory Animal Care (AAALAC) and operates in compliance with the Animal Welfare Act (P.L. 89-544, as amended P.L. 91-578, P, L, 94-279, and P.L. 99-198); the Guide for Care and Use of Laboratory Animals (NIH Publication No. 93-23, 1985 or succeeding revised editions); and the PHSW Policy on Human Care and Use of Laboratory Animals. UTMB has an Animal Welfare Assurance on file (A32314-01) with the Office of Protection from Research Risks. The Animal Resources Center is under the direction of a Doctor of Veterinary Medicine and is staffed by veterinarians with training and expertise in laboratory animal medicine, surgery, clinical care, and diagnostic pathology. Animals are examined daily for feeding grooming and monitored for evidence of illness that would result in euthanasia.

### Removal and storage of the gastrocnemius

Young (3-4 mos), middle aged (13.5mos) and aged (20 mos) WT and DN-p38α^AF/+^ mice were housed in groups of 1-6 per cage, maintained on 12-hour light/dark cycle, with free access to food and water. Immediately after euthanization by CO_2_ the gastrocnemius was excised, immersed in liquid nitrogen and kept at −80°C for Western blot analysis. The opposite lower limb was also removed immersed in 10% neutral buffered formalin and fixed at room temperature for ~5 days. The two parts of the gastrocnemius were then used to measure the maximal gastrocnemius diameter.

### Fluorescenceimmuno-histochemistry

Paraffin-embedded tissue blocks were cut into 5-μm sections in preparation for immunohistochemistry analysis. Histochemistry H&E staining and fluorescence immunohistochemistry were performed as described [58, 59]. The following primary antibodies used for immunofluorescence were purchased from Abcam, Cambridge, MA: beta actin, GAPDH, rabbit anti-ALDH1A1 (1:100, #ab52492,); rabbit anti-ALDH2 (1:200 #ab 108306); mouse anti-BubR1 (1:200, #ab54894); ); rabbit anti-p38α (1:1000, #ab7952); rabbit anti-COX2 (1:200, #ab15191); rabbit anti-protein-HNE (1:500, #HNE11-S, Alpha Diagnostic International, San Antonio, TX); rabbit anti-iNOS (1:500, #ab15323); mouse anti-p16^Ink4a^ (F-12) (1:200, #sc-1661, Santa Cruz Biotech, Dallas, TX); rabbit anti-p19^Arf^ (M-167) (1:200, #sc-1063, Santa Cruz Biotech); and rabbit anti-phosph-p38αMAPK (Thr180/Tyr182) (D3F9) (1:250, #4511, Cell Signaling Technology, Danvers, MA). The sections were visualized using an Olympus BX53 digital microscope. Representative images of interest were acquired using the Olympus CellSense program.

### Immunoblot (Western blot) analyses

Frozen gastrocnemius muscle was directly immersed in cold RIPA lysis buffer and homogenized using an Ultra-Turrax homogenizer and then left on ice for 1 hr. The supernatant was then recovered by centrifugation and protein concentration was determined by the bicinchoninic acid (BCA) technique. Further treatment for Western blot analyses were performed as described [60]. The blots were quantified by NIH ImageJ software.

### Protein normalization

After one-dimensional gel electrophoresis and transfer to nitrocellulose membranes, the post-transfer gels used for each membrane were stained with Gel Code (Pierce, Rockford, IL) and the membranes stained with Ponceau S solution (Sigma-Aldrich) to ensure equal loading. All blots were stripped after probing with the total protein antibodies and probed with anti-β-actin antibody (Sigma-Aldrich) to normalize for loading. The densities of the bands detected by anti-β-actin antibody were measured as described.

### Counting of progenitor cells

H&E-stained sections of gastrocnemius muscles were used to quantify the size of myofiber and the satellite cell density around individual myofibers as described [61]. The myofiber size was calculated and expressed as μm^2^/myofiber, and satellite density as cells/myofiber.

### Measurement of maximal gastrocnemius diameter

The maximal diameter was quantified by measuring the whole area of the H&E-stained cross sections. The H&E-stained sections were visualized under the 2x objective of an Olympus BX53 digital microscope and the measurement was conducted using the “Closed Polygon” function of the Measure module of the Olympus CellSense program. The two sections of each muscle were measured separately and expressed as square millimeters (mm^2^). The two measured values were averaged to represent the animal.

### Statistical analysis

All data are expressed as means ± SEM for at least three independent experiments using at least three mice. Differences between the groups were determined by One-way analysis of variance (ANOVA) with Tukey-Kramer as well as Bonferroni Multiple Comparisons Tests using GraphPad InStat3 software (GraphPad Software Inc., San Diego, CA). Results were considered significant if the p value was less than 0.05.
